# Circulating Levels of MiRNAs From 320 Family in Subjects With Lipodystrophy: Disclosing Novel Signatures of the Disease

**DOI:** 10.3389/fendo.2022.866679

**Published:** 2022-06-06

**Authors:** Alessia Dattilo, Giovanni Ceccarini, Gaia Scabia, Silvia Magno, Lara Quintino, Caterina Pelosini, Guido Salvetti, Roberto Cusano, Matteo Massidda, Lucia Montanelli, Donatella Gilio, Gianluca Gatti, Alessandro Giacomina, Mario Costa, Ferruccio Santini, Margherita Maffei

**Affiliations:** ^1^ Institute of Life Sciences, Scuola Superiore Sant’Anna, Pisa, Italy; ^2^ Obesity and Lipodystrophy Center, Endocrinology Unit, Pisa University Hospital, Pisa, Italy; ^3^ National Research Council, Institute of Clinical Physiology, Pisa, Italy; ^4^ Center for Advanced Studies, Research and Development in Sardinia, Pula (CA), Italy; ^5^ Department of Clinical and Experimental Medicine, Endocrinology Unit, Pisa University Hospital, Pisa, Italy; ^6^ Plastic and Reconstructive Surgery Unit, Hospital of Pisa, Pisa, Italy; ^7^ National Research Council, Institute of Neuroscience, Pisa, Italy; ^8^ Centro Pisano Flash Radiotherapy, Center for Instrument Sharing of the University of Pisa (CPFR@CISUP), Pisa University Hospital, Pisa, Italy

**Keywords:** lipodystrophy subtypes, miRNA, metabolism, gene ontology, circulating biomarker, kobberling

## Abstract

Lipodystrophy (LD) indicates a group of rare disorders, with generalized or partial loss of white adipose tissue (WAT) often associated with metabolic derangements. Heterogeneity/wide spectrum of the disease and lack of biomarkers make diagnosis often difficult. MicroRNAs are important to maintain a correct WAT function and WAT is a source of circulating miRNAs (cmiRs). miRNAs from 320 family were previously detected in the WAT and variably associated to the metabolic syndrome. Our aim was then to investigate if LD can result in altered abundance of cmiRs-320. We collected samples from a cohort of LD subjects of various subtypes and from age matched controls. Use of quantitative PCR determined that cmiRs- 320a-3p, 320b, 320c, 320e are upregulated, while 320d is downregulated in LD. CmiRs-320 power as classifiers was more powerful in the most extreme and defined forms of LD, including the generalized and the Dunnigan subtypes. cmiR-320a-3p showed significant inverse relationships with plasma leptin (P < 0.0001), typically low in LD. The hepatic enzymes gamma-glutamyl transferase (GGT), aspartate aminotransferase (AST), alanine aminotransferase (ALT) and the marker of inflammation C-reactive protein (CRP) were inversely related to cmiR 320d (P < 0.05, for CRP and GGT; P < 0.01, for AST and ALT). Gene ontology analysis revealed cell-cell adhesion as a process regulated by 320 miRNAs targets, thus disclosing a novel route to investigate origin of WAT loss/dysfunction. In conclusion, cmiRs-320 constitute novel biomarkers of LD, abundance of miR320a-3p is inversely associated to indicators related to WAT function, while downregulation of cmiR-320d predicts an altered hepatic profile and higher inflammation.

## Introduction

Lipodystrophy (LD) designates a heterogeneous group of rare disorders, characterized by dysfunction and lack of white adipose tissue (WAT) with a prevalent loss of the subcutaneous (SAT) (1). The spectrum of the disorder is broad and the current classification scheme distinguishes 4 major categories: Congenital Generalized LD (CGL), a very rare disorder with known pathogenetic mechanism due to defects in genes involved in lipid droplet formation (1–3); Familial Partial LD (FPL), the most common form of LD in adults (4); Acquired Partial and Generalized LD (APL and AGL), with variable onset (5) and causes still to be elucidated, although an imbalance in the immune response has been hypothesized (6). Other forms include those associated with progeria, often presenting generalized fat loss (7). Besides, there is a relatively frequent form of LD occurring in human immunodeficiency virus (HIV) infected patients as a consequence of antiretroviral therapy (8), which will not be herein investigated. Metabolic and endocrine derangements, that paradoxically mimic those found in morbid obesity (9), often affect LD subjects: triglyceride overload due to overfeeding or reduced/null capacity to store energy in excess results, in fact, in dysfunctional WAT and consequent dysmetabolism including insulin resistance, dyslipidemia and hepatic steatosis (9, 10). Notwithstanding the heterogeneity of LD, there are considerations that apply to the entire spectrum of the disorder that is worth considering: 1. If we exclude CGL and some forms of FPL (11), for which we have a known cause-effect relationship between gene defects and impaired lipid droplet formation (3), the mechanisms of the other types of LD are to be defined. 2. In most LD patients, especially in the acquired forms, WAT loss is noticed and clinically evaluated when the extension of fat disappearance is advanced and metabolic health compromised, while it would be helpful to diagnose this condition as early as possible.

Among other factors, microRNAs (miRNAs) play a relevant role in the regulation of adipocyte biology (12); loss of function of the miRNA processing enzyme Dicer results in a LD and insulin resistant mouse (13) and adipose tissue-specific ablation of Dgcr8, a key regulator of miRNA biogenesis, results in dysfunctional adipose tissue (14, 15). MiRNAs bind to messenger RNAs, modulate their stability and availability to be translated, their dysregulation being implied in the onset of multiple disorders including cancer, neurodegenerative diseases, and diabetes (16). These small RNAs can be released by cells into the blood flow and reach distant tissues to act as gene expression modulators (17). Of note, WAT releases miRNAs into the circle and analyses conducted in HIV-associated LD revealed a downregulation of WAT expression of Dicer (13), and consistent alterations in the levels of both circulating and adipose tissue miRNAs (cmiRs) (18, 19).

In the present study we chose to focus our attention on 5 miRNAs belonging to the same family, i.e. 320 (from now on referred to as miRs-320), as they present features that are relevant for a better understanding of WAT dysfunction and loss: 1. they were previously reported to be present in the WAT (20) and play a pivotal role in the adipogenic versus osteogenic switch of human mesenchymal stem cells (21); 2. miRs-320 were found to be significantly associated with visceral adipose tissue levels, a depot which is overrepresented in various forms of LD at the expense of SAT loss (22); 3. miRs-320 are overexpressed in metabolically stressed 3T3-L1 murine adipocytes, where they suppress glucose uptake and lipogenesis, while inducing lipolysis *via* the insulin-phosphatidylinositol 3-kinase pathway and endoplasmic reticulum stress signaling (23, 24).

**Figure 1:**
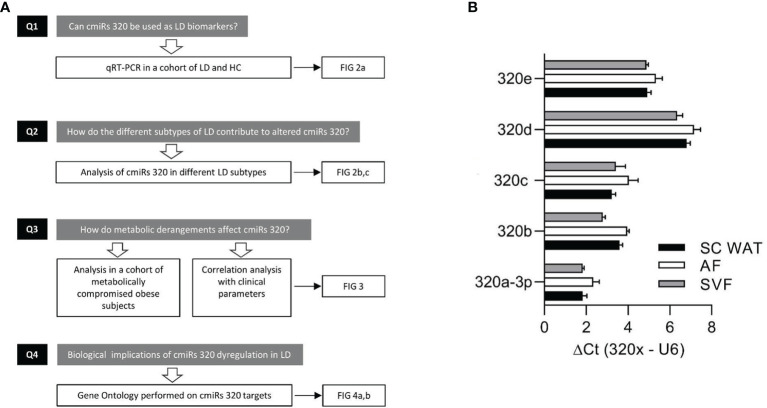
Experimental design and discovery study. **(A)** Flowchart depicting the experimental design consisting in: quantitation of cmiRs-320 levels and further characterization steps as indicated. **(B)** Bar graphs of 320 family miRNAs in subcutaneous WAT and subfractions. Data are expressed as mean ± SEM for 3 independent samples.

In search of novel biomarkers of LD and of molecular mechanisms underlying its pathogenesis and/or downstream targets, we characterized the circulating levels of miRs-320 (cmiRs-320) in subjects affected by the disease considered either as a single heterogeneous cohort or sub-grouped in the various subtypes; we investigated the relationship between cmiRs-320 and the clinical profile, and we finally performed gene ontology search on cmiRs-320 targets to get insights into the biological meaning of our findings. Our experimental design is represented in the flowchart of **Figure 1A**.

## Materials and Methods

### Study Population

Patients attending the outpatient clinic for obesity and lipodystrophies at the University Hospital of Pisa were prospectively enrolled in the study. Medical history, clinical and laboratory data were collected and anthropometric measures were taken by standardized methods throughout the study. We recruited: patients with LD, the diagnosis of LD sub-type was made based on the published criteria (2, 25); obese subjects with a BMI > 35 Kg/m2 associated to at least 2 metabolic derangements; healthy controls (HC) recruited among subjects undergoing routinary endocrinological tests with no comorbidities. Eligible were all patients between 2 and 80 years old and criteria for exclusion were presence of malignancy or diseases that may influence miRNA expression (26). A subgroup of controls, herein defined adult healthy controls (AHC), was selected for a proper age matching with FPLD1 subjects (see also **Supplementary Table 2**). Patients’ recruitment and informed consent were carried out in accordance with approved guidelines from the Local Ethical Committee (CEAVNO, Protocol number D 19440).

### Human Samples

Plasma: Whole blood was collected into EDTA tubes (BD Vacutainer, Franklin Lakes NJ, USA) and plasma obtained by centrifugation at 3000 rpm, 4°C, 15 minutes, prior to a second centrifugation at 12600 g, 4°C for 10’ and storage at −80°C for subsequent analysis. Aliquots of plasma from the samples were thawed only once. We estimated cell and hemolysis contamination in our plasma samples by: 1. eye judgement checking the sample against a colorimetric scale, 2. comparing the levels of a miRNA highly expressed in red blood cells (hsa-miR-451a) and one unaffected by hemolysis (hsa-miR-23a-3p) (27). In all our samples we have found the ΔCt (miR-23a-3p - miR-451a) is smaller than five, showing the absence of hemolysis.

### WAT Samples and Isolation of Adipocyte Fraction (AF) and Stromal Vascular Fraction (SVF)

Biopsies of SAT were obtained during elective surgery and one aliquot promptly frozen into liquid nitrogen and stored at -80°C. Fresh SAT was sub-fractionated into SVF and AF as previously described (28). Briefly, the subcutaneous WAT biopsy obtained during elective abdominal surgery was cut in little pieces and incubated 1 hour at 37°C with 2 mg/ml collagenase Type 1 (Sigma-Aldrich, St. Louis, MO, USA). The digestion was stopped by adding 3 volumes of DMEM completed with 10% FBS, then the homogenate was filtered through a 100 “mesh. The filtrate was then centrifuged 1,000 g for 5 min at RT in order to separate AF in the upper phase and SVF in the lower phase. The two cell fractions were separately collected in different tubes for further processing.

### RNA Isolation, cDNA Synthesis and MiRNA Quantification

cmiRs were extracted from 200 µL aliquots of plasma using a miRNeasy Serum/Plasma Kit (Qiagen, Hilden, Germany), following the manufacturer’s protocol. During the extraction process 3.5 μl (1.6 × 108 copies/μl) of C. elegans’miR-39 miRNA mimic spike-in control (cel-miR-39) was added to monitor the RNA isolation before purification. miRNAs were isolated from tissues using a miRNeasy micro Kit (Qiagen, Hilden, Germany) following the manufacturer’s protocol. The quality and integrity of RNA samples were evaluated using a RNA 6000 Nano LabChip on an Agilent 2100 Bioanalyzer (Santa Clara, CA, USA). RNA eluted in RNase-free water was stored at −80°C until use.

3.75ul of RNA extracted from plasma and 500ng of RNA extracted from cells were reverse transcribed using Mir-X miRNA First-Strand Synthesis Kit (Takara, Mountain View, California, USA) following the manufacturer’s protocol. cDNA templates were then diluted 4X and 20X respectively in nuclease free water and 1ul were used in qPCR reactions. qPCR was performed using SYBR Green-based technology.

For normalization of miRNA expression: in plasma samples the exogenous spike-in control cel-miR-39 was measured by means of cel-miR-39 miScript Primer Assays (Qiagen); in all the other samples the expression of small nuclear RNA U6 served as control of equal loading (U6 Forward and Reverse Primers from Takara).

The ΔΔCt comparative method was used for data analysis and measurements with cycles greater than 36 were considered undetectable. miRNA values were expressed using the following procedure: in each case the mean of the control group was calculated and each value was then divided by that number and multiplied by 100.

A list of forward primers used for qPCR detection and their sequence is provided below.

hsa-miR-320a-3p 5’-AAAAGCTGGGTTGAGAGGGCGA-3’

hsa-miR-320b 5’-AAAAGCTGGGTTGAGAGGGCAA-3’

hsa-miR-320c 5’-AAAAGCTGGGTTGAGAGGGT-3’

hsa-miR-320d 5’-AAAAGCTGGGTTGAGAGGA-3’

hsa-miR-320e 5’-AAAGCTGGGTTGAGAAGG-3’

The reverse primer (mRQ) was included in the Mir-X™ miRNA First-Strand Synthesis Kit (Takara, #638313) and its sequence is patent pending.

### Gene Ontology Analysis

Target gene prediction for 320 miRNAs was performed using miRDB with a target score cut off set at 60. The identified proteins were ranked according to their prediction score and analyzed through Gene Ontology enrichment analysis and visualization tool GOrilla, publicly available as a web-based application at: http://cbl-gorilla.cs.technion.ac.il, which uses a statistical model that supports the discovery of GO terms enriched at the top of a ranked list, enabling a threshold to be determined in a data driven manner and providing an exact p-value for the observed event. GOrilla output is a series of GO terms associated to a P value, an FDR and an enrichment score, where p-value for the observed enrichment is calculated taking threshold multiple testing into account; FDR q-value is the correction of the above p-value for multiple testing using the Benjamini and Hochberg (1995) (29) method.

Enrichment (N, B, n, b) is defined as follows:

N - is the total number of genes

B - is the total number of genes associated with a specific GO term

n - is the number of genes in the top of the user’s input list or in the target set when appropriate

b - is the number of genes in the intersection

Enrichment=(b/n)/(B/N) (30)

IPA (Qiagen, Hilden, Germany) was employed to establish the network among proteins (as identified by GOrilla) and to graphically elaborate this information.

### Laboratory Tests

Venous blood samples were obtained after an overnight fasting for measurement of serum glucose, HbA1c, triglycerides, total cholesterol, LDL-C, HDL-C, CRP, ALT, AST, GGT. Serum leptin was measured by enzyme-linked immunosorbent assay (ELISA) (Mediagnost, Germany).

### Statistics

Statistical analysis was performed using GraphPad Prism 8 software. Student’s t-test or Mann–Whitney test were used for pair comparisons for normally or not normally distributed data, as appropriate. Parametric One-way ANOVA followed by Bonferroni *post-hoc* test or Kruskal-Wallis followed by Dunn’s Multiple Comparison test were respectively used for normally or not normally distributed data. Non-parametric correlation analysis (Spearman) was performed. P<0.05 was considered statistically significant.

## Results

### MiRs From 320 in LD

To substantiate the rational of our study, i.e. analysis of miRs-320 in LD, we first wanted to confirm their expression in WAT. To this end we used biopsies from 3 subjects for whom we could establish the presence of miRs-320 in intact SAT and in both the Adipocyte and Stromal Vascular Fraction (AF and SVF). As a measure of miRNA abundance for each sample we calculated the delta Ct between the miRNA of interest and U6, a small nuclear RNA, that for its stability and relatively high expression, is widely used as internal reference gene for miRNA quantification (31): as shown in **Figure 1B** delta Cts are always <8, indicating that both WAT fractions may be sources of miRs-320. We next wanted to better characterize their expression in LD.

**Figure 2:**
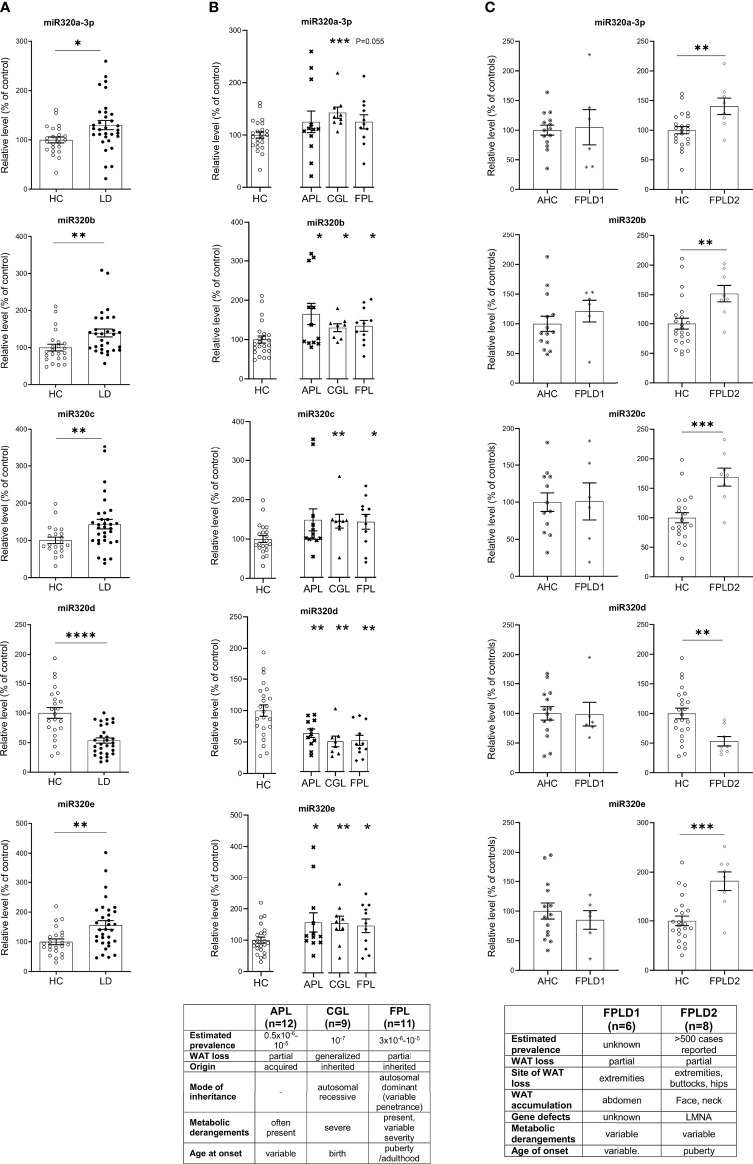
Relative level of cmiRs-320 in LD and HC subjects. **(A)** Scatter plots with error bar of cmiRs 320 in a) LD (n = 32) versus HC (n = 23). **(B)** in APL (n = 12), CGL (n = 9), FPL (n = 11) versus HC. Each asterisk denotes a pairwise comparison of 2 groups. **(C)** FPLD1 (*n* = 6) versus an *ad-hoc* selected group of age matched controls Adult Healthy Controls (AHC) (*n* = 14) and FPLD2 (*n* = 8) versus HC. Data, expressed as percentage of the mean of HC or AHC (in the case of FPLD1), are shown as mean ± SEM (standard error of the mean). *P < 0.05, **P < 0.01, ***P < 0.001, ****P < 0.0001, by Student’s t test or Mann-Whitney as appropriate.

MiRs-320 were then analyzed in the plasma of patients with or without LD. As shown in **Figure 2A** studies conducted by quantitative PCR in a cohort of healthy controls and affected patients (23 HC vs 32 LD, see clinical characteristics in **Table 1**) proved that cmiRs 320 were differentially expressed in LD versus HC. In the case of miR-320d the circulating levels were lower in LD as compared to HC; in all the other cases (miR-320a-3p, 320b, 320c, 320e) there was a significant upregulation. Corresponding delta Ct values are also reported in **Supplementary Figure 1** (**SF 1**). To evaluate the relevance of the 5 miRNAs as biomarkers of LD, we conducted an analysis of Receiver Operating Characteristic (ROC) that revealed an area under the curve (AUC) ranging from 0.71 to 0.81, with p-values between 0.0001 and 0.007 (**SF 2A**). To improve the power of the classifier, we tried several combinations of the miRNAs delta Cts (Cts miR of interest – Cts Cel-miR-39) as a possible discriminating measure between HC and LD: the sum of the measure concerning miR-320a-3p, miR-320b, miR-320c and miR-320e was the best solution providing an AUC of 0.85 (P < 0.0001). The optimal cut-off point of the combination was determined as being ≤24.48 (sensitivity: 75.00; specificity: 85.71) (**SF 2B**).

**Table 1:** Physical and clinical parameters of subjects enrolled in the study*.

Physical and clinical parameters	Controls (*N* = 23; F = 16)	LD (w/o FPLD1) (*N* = 32; F = 24)	Obese (*N* = 13; F = 8)	Multiple Comparisons Result
Age (years)	34.5 ± 3.9	35.0 ± 3.4	49.1 ± 3.5	ns
BMI (Kg/m^2^)	21.2 ± 0.6	20.6 ± 0.7	43.5 ± 1.7	a ns; b ****; c ****
Glucose (mg/dL)	91.0 ± 2.0	94.0 ± 3.3	111.7 ± 7.3	ns
TG (mg/dL)	80.8 ± 9.2	153.6 ± 27.3	154.1 ± 13.4	a **; b ***; c ns
Chol (mg/dL)	180 ± 7.6	169.8 ± 5.7	198.8 ± 10.0	a ns; b ns; c *
HDL-C (mg/dL)	65.4 ± 3.7	45.25 ± 2.7	47.5 ± 3.2	a ***; b *; c ns
LDL-C (mg/dL)	106.3 ± 7.1	113.7 ± 5.2	137.7 ± 8.7	a ns; b *; c ns
CRP (mg/L)	0.05 ± 0.01	0.7 ± 0.2	0.88 ± 0.16	a **; b ****; c ns
AST/GOT (U/L)	18.8 ± 0.8	28.1 ± 2.1	36.4 ± 7.3	a**; b*; c ns
ALT/GPT (U/L)	13.5 ± 1.0	34.4 ± 4.6	55.9 ± 13.3	a****; b****; c ns
GGT (U/L)	11.8 ± 1.4	28.4 ± 4.1	59.3 ± 16.0	a **; b****; c ns
LD Subtype		11 FPL (of which 8 FPLD2) 12 APL 9 CGL		

a = Controls vs. LD; b= Controls vs. Obese; c = LD vs. Obese. *P < 0.05, **P < 0.01, ***P < 0.001, ****P < 0.0001. Data are expressed as Mean ± SEM. ns, not significant.

We then wanted to investigate if in the different subtypes included in the large cohort of LD patients the differences in cmiRs 320 were confirmed. To this end we considered 3 subtypes: CGL, FPL and APL. For all the comparisons we confirmed differences with the same sign found in whole cohort which in most cases (except 2) were significant. Within these 3 categories, the capacity of cmiRs-320 to act as disease classifier is more powerful for CGL (**Figure 2B**; **Supplementary Table 1**, **ST 1**). Multiple comparison analysis (one-way ANOVA) revealed no significant differences among LD subtypes for cmiRs-320. In the analyses presented so far we did not include subjects with Familial Partial LD, type 1 (FPLD1 or Köbberling): the reason is that they show significantly higher age and BMI compared to the rest of the LD cohort and required an *ad-hoc* group of properly matched controls (Adult Healthy Controls, AHC, clinical characteristics of FPLD1 and AHC in **ST 2**). In **Figure 2C** the analysis of cmiRs-320 in FPLD1 subjects are reported next to the analysis for FPLD2, or Dunnigan. As for FPLD1 subtype, there is no specific candidate gene and a polygenic mode of inheritance has been postulated (32) for a phenotype with a wide spectrum and consequent challenging diagnosis, that presents with reduction of fat in the extremities and accumulation in the central part of the body, similar to visceral obesity. FPLD2, on the other hand, collects individuals with a defect in the gene coding for *lamin A/C*, characterized by dramatic loss of fat at the extremities/trunk and typical accumulation around the neck (33). Symptoms manifestation usually starts after puberty (4), metabolic derangements are present with variable degree of severity. Of interest, all cmiRs-320 significantly discriminate FPLD2 subjects from HC; conversely FPLD1 subjects are overlapping with AHC in all cases (**Figure 2C**; **ST 1**).

Alltogether these results indicate cmiRs-320 as an additional tool to mark the LD disorder and reiterate the importance of considering each LD subtype as a different condition of WAT dysfunction.

### Relationship Between CmiRs-320 and Metabolism

**Figure 3:**
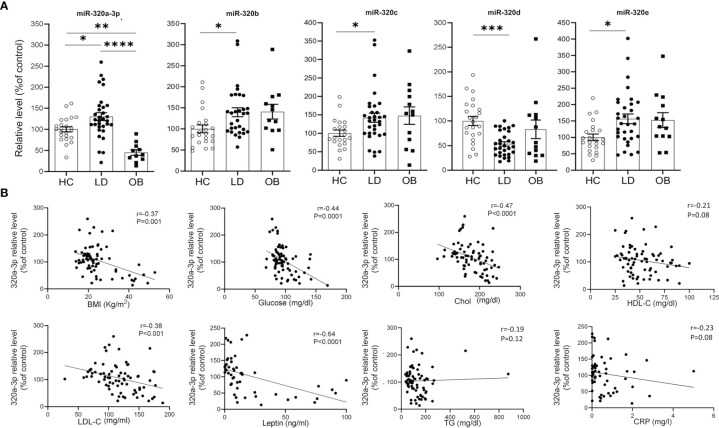
Relationship between cmiRs-320s and metabolism. **(A)** Scatter plots with bar of circulating miRNAs from the 320 family in HC, LD and OB subjects. All data are expressed as percentage of the mean of HC and are shown as mean ± SEM. *P < 0.05, **P < 0.01, ***P < 0.001 and ****P < 0.0001 by one-way ANOVA or Kruskal-Wallis, as appropriate. **(B)** Correlation analysis between BMI, glucose, total cholesterol, HDL-C, LDL-C, plasma leptin, triglycerides, CRP and cmiRs-320a-3p in the total population (LD + OB + HC). Spearman’s r and relative p-value are indicated.

Our effort was then to understand the role of the metabolic derangements often affecting LD subjects in driving cmiRs-320 dysregulation. As dysmetabolism found in LD reminds that associated to obesity, we analyzed the concentration of cmiRs-320 in the plasma of a cohort of subjects with obesity (OB), displaying lipid and hepatic profiles similar to those with LD (**Table 1**), and we reasoned that if altered metabolism drives cmiRs-320 abundance, similar changes should be observed in LD and OB with respect to HC. In 3 cases (320b, c, e) the OB group shows a trend similar to LD, when compared to HC, albeit no statistically significant; in the case of miRs-320a-3p we found a change of opposite sign in LD and OB (**Figure 3A**). As a second approach we investigated the relationship between cmiRs-320 and clinical parameters relevant in defining the metabolic profile. Correlation analysis performed for the total population (LD + OB + HC) indicates that miR320a-3p shows the highest number of significant associations and it is inversely related to BMI, glucose, HbA1c, total cholesterol, low density lipoprotein-cholesterol (LDL-C) and leptin (**Figure 3B** and **Table 2**), whereas miR320d is the only member of family 320 showing significant and inverse relationships with the 3 hepatic enzymes gamma-glutamyl transferase (GGT), aspartate aminotransferase (AST), alanine aminotransferase (ALT), and with C-reactive protein (CRP). Similar analyses were performed for all the other miRNAs and the results are shown in **Table 2**.

**Table 2:** Results of correlation analysis for clinical parameters with relative levels of cmiRs-320.

Names and metric units of clinical parameters	320a-3p	320b	320c	320d	320e
**BMI (Kg/m^2^)**	-0.37**	ns	ns	ns	ns
**Glucose (mg/dl)**	-0.44***	ns	-0.29*	ns	-0.25*
**HbA1c (mmol/mol)**	-0.37*	-0.29*	ns	ns	-0.36*
**TG (mg/dl)**	ns	ns	ns	ns	ns
**Chol (mg/dl)**	-0.47****	ns	-0.24*	ns	-0.32**
**HDL-C (mg/dl)**	ns	-0.32**	ns	ns	-0.24*
**LDL-C (mg/dl)**	-0.38***	ns	ns	ns	ns
**Leptin (ng/ml)**	-0.64****	ns	ns	ns	ns
**CRP (mg/dl)**	ns	ns	ns	-0.33*	ns
**GGT (U/L)**	ns	ns	ns	-0.24*	ns
**AST/GOT (U/L)**	ns	ns	ns	-0.35**	ns
**ALT/GPT (U/L)**	ns	ns	ns	-0.35**	-0.25*

Correlation analysis was assessed in the total population (LD + OB + HC). The Table reports Spearman r and *P < 0.05, **P < 0.01, ***P < 0.001, ****P < 0.0001, ns significant.

### Gene Ontology and Pathway Analysis

**Figure 4:**
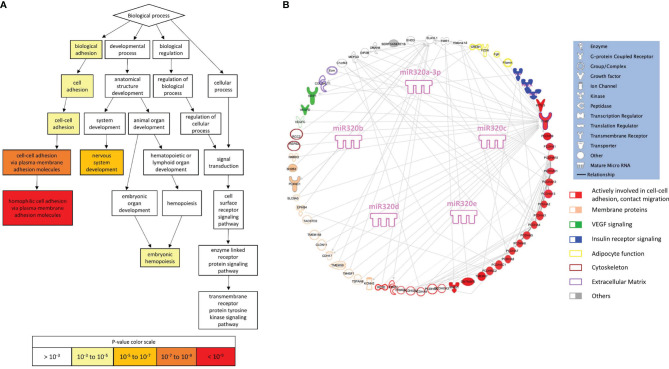
Gene ontology and pathway analysis. **(A)** Visualization of enriched GO terms for a ranked list of 320 miRNA targets (from the publicly available platform GOrilla). The white to red scale indicates progressively increased significance of the enrichment. **(B)** Graphical representation of the network generated by the proteins included in the enriched GO terms (elaborated using IPA, Qiagen, Hilden, Germany).

To search for a biological significance of these findings and investigate the involvement of specific regulatory networks, we performed the *in silico* prediction of target genes possibly modulated by miRNA 320s by integrating the use of the publicly available applications miRDB and TargetScan. Gene ontology (GO) enrichment analysis was performed on targets by using GOrilla, a web-based application, that identifies enriched GO terms for biological processes in ranked lists of genes. Highly significant GO terms associated to biological processes were identified, including: homophilic cell adhesion *via* plasma membrane adhesion molecules (P = 5.97E-10, false discovery rate, FDR, 3.93E-6); cell-cell adhesion *via* plasma membrane adhesion molecules (P = 6.31E-8, FDR 2.08E-4); nervous system development (P = 8.82E-6, FDR 1.93E-2); cell-cell adhesion (P = 1.66E-5, FDR 2.74E-2); biological adhesion (P = 2.89E-5, FDR 3.8E-2). In **Figure 4A** a graphical output of this result. A comprehensive Table showing the detail of these *in silico* results is shown as **ST 3**. **Figure 4B** shows the 320 miRNAs target proteins interaction network. The classes of proteins mostly represented are membrane and adhesion proteins: among others, gamma protocadherins constitute a substantial proportion of the protein forming the network and are highly interrelated.

*In silico* analysis suggests that miRs-320 target proteins which are highly aggregated in defining the capacity of cells to build contacts, and strengthen the architecture of tissues.

## Discussion

MiRNAs are necessary to maintain WAT homeostasis as demonstrated in experimental animal models, and LD conditions associated to HIV result in their altered production and release (13, 18). Based on these lines of evidence we wanted to investigate if in different forms of human LD, of genetic or acquired origin, with partial or total loss of WAT, signatures of the disease may be tracked in the blood as variations in the level of circulating miRNAs. Indeed, we found a dysregulation of the miR-320 family, reportedly present in WAT (24) and implied as permissive for adipogenesis of human mesenchymal stem cells (34), however not previously associated to *in vivo* conditions of adipocyte dysfunction. In this regard it is worth observing that when plasma miRnome profiling was conducted in subjects with HIV-associated LD *versus* controls, cmiRs 320 were not identified as dysregulated (35). Even if both associated to fat loss the HIV-related and not related forms of LD are profoundly different; in addition, subjects enrolled in that study did not show significant metabolic alterations, differently from our cohort. Furthermore, our findings suggest that cmiRs 320 discriminate more powerfully LD subtypes with a known genetic origin and defined phenotype (i.e. CGL and FPLD2) than forms with an unknown origin and subtle phenotype: it is thus somewhat expected that in HIV-associated forms of LD, highly variable in terms of fat loss and redistribution, cmiRs 320 may not be significantly dysregulated.

Interestingly, we found that miRs-320 a3p, b, c and e are elevated, whereas d is reduced in LD. While we are not able to predict the consequences of this opposite regulation, it is worth mentioning that in at least one case, miRNA family 200, different members have opposite effects on their target *c-JUN*, with miR-200a inhibiting its production and miR-200b acting in non-canonical way and inducing its expression (36): we cannot exclude a similar scenario for miRNAs 320. Elaborating on the same subject we also have to consider that the five 320 members (a-e), are coded by loci in different chromosomes (chr) (320a, chr8; 320b, chr1; 320c, chr18; 320d, chr13 and chrX; 320e, chr19 and therefore their transcription is under the control of 6 independent cis regulatory elements: of note miR-320d is the only coded by 2 loci in 2 different chromosomes (X and 13) (37, 38).

Despite the high degree of speculation that elaborating on altered concentrations of circulating miRNAs may involve, we will herein discuss different levels of direct and less direct implications suggested by the LD-related changes in the cmiRs-320.

The first level stems from the use of cmiRs-320 as novel biomarkers of LD. The power to discriminate between affected and not affected individuals becomes even stronger for the specific subtypes CGL and FPLD2, that, although different in terms of extension of fat loss, share the certainty of the diagnosis based on well-defined phenotypes and genetic testing. At the other extreme FPLD1, for which cmiRs-320 seem totally useless as classifier. Despite recurrent in families, a genetic cause of FPLD1 has yet to be identified and subjects described in previous studies are usually women in their mature age, characterized by peripheral LD, and one or more of the following features: central obesity and components of the metabolic syndrome, including diabetes, hypertension, and presence of hypertriglyceridemia (39). Consistent with this description the average BMI of FPLD1 subjects participating to this study defines them as overweight. Compared to the other subtypes, FPLD1 presents then with a more subtle phenotype, which in the clinical setting may partly overlap with the physiological loss of gluteofemoral fat frequent upon aging (40) and, for other aspects, with metabolically complicated obesity (32, 41). In summary, the discrepancy with the other LD subtypes emerged from the present analysis of cmiRs-320 is not a *unicum* but adds up to other features in describing FPLD1 as an atypical (less defined) form of LD.

The second question is whether cmiRs 320 differential level is downstream of metabolic derangements present in LD, that mirror obesity-associated metabolic syndrome. The fact that for 1 out of 5 miRNAs (320a-3p) the sign of the regulation with respect to control is opposite in LD and OB suggests that dysmetabolism may contribute, but is not sufficient to explain the change. One other way to elaborate on this aspect is to evaluate if metabolic syndrome signatures of LD subjects, namely, higher TG, CRP and hepatic enzymes and lower HDL-C, consistently associate to the sign of change of cmiRs-320. Given that cmiRs-320, except d, are higher in LD we would expect a positive association with metabolic/inflammatory parameters if the latter served as a trigger for their elevation. What we found, instead, is that where present, significant relationships were inverse. There are 2 cases, however, in which we could hypothesize an impact of dysmetabolism on cmiRs. These are represented by the relationship between cmiR-320b negatively associated to HDL-C, and that between cmiR-320d, which, being downregulated in LD, is inversely associated to the concentration of hepatic enzymes and of CRP. In line with our results, lower level of serum exosomal miR-320d was found in hepatocellular carcinoma (42). Based on these evidences, dyslipidemia seems then to moderately contribute to the LD associated dysregulation of cmiR-320 b, while liver dysfunction and inflammation, abundantly interrelated conditions (43), seem to predict the downregulation of cmiR-320d.

Of note miR-320a-3p, besides showing the highest number of inverse relationships with clinical parameters, is also inversely related to BMI and plasma leptin. Notwithstanding the tight relationship between the 2 parameters (44), to consider is that both reflect WAT mass, while leptin is also tightly related to WAT function (44, 45). Circulating levels of miR-320a-3p predict therefore the salient aspects of the disease, i.e. reduced WAT mass and function.

A third interesting level of speculation emerges from gene ontology search, carried out on miRs-320 targets: there are in fact strong and highly significant indications that cell-cell adhesion and migration are modulated by proteins targeted by these miRNAs, including integrin 1 beta (ITG1B) and several protocadherins. Noteworthy, the expression of ITG1B in WAT is positively associated with body fat mass in humans (46), consistent with a putative downregulation in LD, possibly triggered by the upregulation of miRs-320. Integrins, which act also as receptors and constitute the most well studied molecules in the organization of the extra cellular matrix (ECM) (47), reside at the cell surface and require inside-out activation to reach a higher affinity for the ECM components. This activation process is facilitated by binding of intracellular adaptor proteins like Kindlin. ITG1B deletion specifically in adipocytes reduced capacity to increase WAT mass in mice exposed to high fat diet (46). Even more compelling evidences indicate *kindlin* as implied in a correct WAT function, as its deletion in the adipocyte leads to a mouse model with LD and severe metabolic alterations (48). These findings and our *in silico* data stimulate considerations on the role that an impaired cell-cell adhesion, migration and cross talk might have in the onset of human LD, a condition in which the WAT loses its architecture and undergoes a process of deaggregation and sometimes redistribution. Ground to this hypothesis is provided by the decrease in the expression of many genes involved in cell adhesion observed in 3T3-L1 adipocytes treated with ritonavir and lopinavir (49), two protease inhibitors used to treat HIV infected patients and implied in the onset of the HIV-associated form of LD (50).

This study has some limitations and questions that remain to be addressed: 1. relatively small number of recruited patients, due to the low prevalence of LD and of its subtypes; 2. no direct biological evidence to link cmiRs-320 dysregulation with LD pathogenesis and 3. The source/s of dysregulated cmiRs-320 are to be elucidated. Although they may originate from dysfunctional WAT, it is counterintuitive to reconcile increase of cmiRs-320 with dramatic fat loss, for instance in the CGL subtype. Our data, however, suggest that the stromovascular component of the adipose tissue, preserved also in the extreme generalized forms of LD (51, 52), may contribute to their release and availability of WAT biopsies from LD subjects will be essential to get insights into this matter.

In conclusion, altered abundance of circulating levels of miRNAs from 320 family constitutes a novel signature of lipodystrophy. cmiRs-320 are inversely associated with several components of the metabolic syndrome and higher abundance of cmiR-320a-3p, typical of LD, predicts lower plasma leptin. *In silico* results indicate that targets of miRs-320 influence cell-cell adhesion, thus suggesting this process as a novel direction to investigate the origin of LD.

## Data Availability Statement

The original contributions presented in the study are included in the article/**Supplementary Material**. Further inquiries can be directed to the corresponding author.

## Ethics Statement

The studies involving human participants were reviewed and approved by CEAVNO, protocol number D 19440 . Written informed consent to participate in this study was provided by the participants’ legal guardian/next of kin.

## Author Contributions

Conceptualization and study design, MMaf. Methodology, MMaf, AD, GSc, RC, MMas, and MC. Investigation, AD, GSc, LQ, and MC. Formal analysis, MMaf, AD, and GSc. Resources, MMaf, FS, AD, GC, SM, GSa, GG, AG, CP, LM, DG, and GSc. Writing original draft preparation, MMaf. Writing review and editing, AD, GSc, GC, FS, and MC. Supervision, MMaf and MC. Funding acquisition, MMaf, AD, and FS. All authors contributed to the article and approved the submitted version.

## Funding

This research was funded by Italian Ministry of Education, University and Research, Project code 2017L8Z2EM to FS.

## Conflict of Interest

The authors declare that the research was conducted in the absence of any commercial or financial relationships that could be construed as a potential conflict of interest.

## Publisher’s Note

All claims expressed in this article are solely those of the authors and do not necessarily represent those of their affiliated organizations, or those of the publisher, the editors and the reviewers. Any product that may be evaluated in this article, or claim that may be made by its manufacturer, is not guaranteed or endorsed by the publisher.

## Abbreviations

LD, Lipodystrophy/subjects with Lipodystrophy; HC, healthy controls; AHC, adult healthy controls; OB, subjects with obesity; WAT, white adipose tissue; SAT, subcutaneous adipose tissue; VAT, visceral adipose tissue; miRNAs, microRNAs; cmiRNAs, circulating microRNAs; cmiRs 320, circulating miRNAs of the 320 family; GL, generalized Lipodystrophy; AGL, acquired generalized Lipodystrophy; CGL, congenital generalized Lipodystrophy; PL, partial Lipodystrophy; FPL, familial partial Lipodystrophy; APL, acquired partial Lipodystrophy; AF, adipose fraction; SVF, stromal vascular fraction; cel-miR-39, *Caenorhabditis elegans*-miR-39; TG, triglycerides; Chol, total cholesterol; HDL-C, high density lipoprotein-cholesterol; LDL-C, low density lipoprotein-cholesterol; CRP, C-reactive protein; AST, aspartate aminotransferase; GOT, glutamic oxaloacetic transaminase; ALT, alanine aminotransferase; GPT, glutamic pyruvic transaminase; GGT, gamma-glutamyl transferase; ROC, Receiver Operating Characteristic; AUC, area under the curve; CI, confidence interval; Ct, cycle threshold; GO, gene ontology; ECM, Extra Cellular Matrix; RUNX2, RUNX family transcription Factor 2; ITG1B, integrin 1 beta.

## References

[1] Brown RJ Araujo-Vilar D Cheung PT Dunger D Garg A Jack M. The Diagnosis and Management of Lipodystrophy Syndromes: A Multi-Society Practice Guideline. J Clin Endocrinol Metab (2016) 101(12):4500–11. doi: 10.1210/jc.2016-2466 PMC5155679 27710244

[2] Araújo-Vilar D Santini F. Diagnosis and Treatment of Lipodystrophy: A Step-by-Step Approach. J Endocrinol Invest (2019) 42(1):61–73. doi: 10.1007/s40618-018-0887-z 29704234 PMC6304182

[3] Xu S Zhang X Liu P. Lipid Droplet Proteins and Metabolic Diseases. Biochim Biophys Acta - Mol Basis Dis (2018) 1864(5):1968–83. doi: 10.1016/j.bbadis.2017.07.019 28739173

[4] Akinci B Onay H Demir T Savas-Erdeve Ş Gen R Simsir IY. Clinical Presentations, Metabolic Abnormalities and End-Organ Complications in Patients With Familial Partial Lipodystrophy. Metabolism (2017) 72:109–19. doi: 10.1016/j.metabol.2017.04.010 28641778

[5] Misra A Garg A. Clinical Features and Metabolic Derangements in Acquired Generalized Lipodystrophy. Med (Baltimore) (2003) 82(2):129–46. doi: 10.1097/00005792-200303000-00007 12640189

[6] Garg A Misra A. Lipodystrophies: Rare Disorders Causing Metabolic Syndrome. Endocrinol Metab Clin North Am (2004) 33(2):305–31. doi: 10.1016/j.ecl.2004.03.003 15158521

[7] Özen S Akıncı B Oral EA. Current Diagnosis, Treatment and Clinical Challenges in the Management of Lipodystrophy Syndromes in Children and Young People. J Clin Res Pediatr Endocrinol (2020) 12(1):17–28. doi: 10.4274/jcrpe.galenos.2019.2019.0124 31434462 PMC7127888

[8] Hussain I Garg A. Lipodystrophy Syndromes. Endocrinol Metab Clin North Am (2016) 45(4):783–97. doi: 10.1016/j.ecl.2016.06.012 PMC7590232 27823605

[9] Lim K Haider A Adams C Sleigh A Savage D. Lipodystrophy: A Paradigm for Understanding the Consequences of “Overloading” Adipose Tissue. Physiol Rev (2020) 101:907–93. doi: 10.1152/physrev.00032.2020 33356916

[10] Cinti S. The Adipose Organ at a Glance. Dis Model Mech (2012) 5(5):588–94. doi: 10.1242/dmm.009662 PMC3424455 22915020

[11] Patni N Li X Adams-Huet B Vasandani C Gomez-Diaz RA Garg A. Regional Body Fat Changes and Metabolic Complications in Children With Dunnigan Lipodystrophy-Causing LMNA Variants. J Clin Endocrinol Metab (2019) 104(4):1099–108. doi: 10.1210/jc.2018-01922 PMC6382455 30418556

[12] Sun L Xie H Mori MA Alexander R Yuan B Hattangadi SM. Mir193b-365 is Essential for Brown Fat Differentiation. Nat Cell Biol (2011) 13(8):958–65. doi: 10.1038/ncb2286 PMC3149720 21743466

[13] Mori MA Thomou T Boucher J Lee KY Lallukka S Kim JK. Altered miRNA Processing Disrupts Brown/White Adipocyte Determination and Associates With Lipodystrophy. J Clin Invest (2014) 124(8):3339–51. doi: 10.1172/JCI73468 PMC4109560 24983316

[14] Xu D Sun L. Role of microRNA Biogenesis in Adipocyte and Lipodystrophy. Adipocyte (2015) 4(3):222–4. doi: 10.1080/21623945.2014.995507 PMC4497299 26257995

[15] Kim H-J Cho H Alexander R Patterson HC Gu M Lo KA. MicroRNAs Are Required for the Feature Maintenance and Differentiation of Brown Adipocytes. Diabetes (2014) 63(12):4045–56. doi: 10.2337/db14-0466 PMC4238002 25008181

[16] Chen X Ba Y Ma L Cai X Yin Y Wang K. Characterization of microRNAs in Serum: A Novel Class of Biomarkers for Diagnosis of Cancer and Other Diseases. Cell Res (2008) 18(10):997–1006. doi: 10.1038/cr.2008.282 18766170

[17] Valadi H Ekström K Bossios A Sjöstrand M Lee JJ Lötvall JO. Exosome-Mediated Transfer of mRNAs and microRNAs Is a Novel Mechanism of Genetic Exchange Between Cells. Nat Cell Biol (2007) 9(6):654–9. doi: 10.1038/ncb1596 17486113

[18] Thomou T Mori MA Dreyfuss JM Konishi M Sakaguchi M Wolfrum C. Adipose-Derived Circulating miRNAs Regulate Gene Expression in Other Tissues. Nature (2017) 542(7642):450–5. doi: 10.1038/nature21365 PMC5330251 28199304

[19] Squillace N Bresciani E Torsello A Bandera A Sabbatini F Giovannetti C. Changes in Subcutaneous Adipose Tissue microRNA Expression in HIV-Infected Patients. J Antimicrob Chemother (2014) 69(11):3067–75. doi: 10.1093/jac/dku264 25063777

[20] Jiménez-Lucena R Rangel-Zúñiga OA Alcalá-Díaz JF López-Moreno J Roncero-Ramos I Molina-Abril H. Circulating miRNAs as Predictive Biomarkers of Type 2 Diabetes Mellitus Development in Coronary Heart Disease Patients From the CORDIOPREV Study. Mol Ther - Nucleic Acids (2018) 12:146–57. doi: 10.1016/j.omtn.2018.05.002 PMC6023857 30195754

[21] Cho WCS. MicroRNAs: Potential Biomarkers for Cancer Diagnosis, Prognosis and Targets for Therapy. Int J Biochem Cell Biol (2010) 42(8):1273–81. doi: 10.1016/j.biocel.2009.12.014 20026422

[22] Munetsuna E Yamada H Ando Y Yamazaki M Tsuboi Y Kondo M. Association of Subcutaneous and Visceral Fat With Circulating microRNAs in a Middle-Aged Japanese Population. Ann Clin Biochem Int J Lab Med (2018) 55(4):437–45. doi: 10.1177/0004563217735124 28920467

[23] Ling H-Y Ou H-S Feng S-D Zhang X-Y Tuo Q-H Chen L-X. CHANGES IN microRNA (Mir) PROFILE AND EFFECTS OF miR-320 IN INSULIN-RESISTANT 3t3-L1 ADIPOCYTES. Clin Exp Pharmacol Physiol (2009) 36(9):e32–9. doi: 10.1111/j.1440-1681.2009.05207.x 19473196

[24] Liu L Li X. Downregulation of miR-320 Alleviates Endoplasmic Reticulum Stress and Inflammatory Response in 3T3-L1 Adipocytes. Exp Clin Endocrinol Diabetes (2021) 129(02):131–7. doi: 10.1055/a-1012-8420 31634961

[25] von Schnurbein J Adams C Akinci B Ceccarini G D’Apice MR Gambineri A. European Lipodystrophy Registry: Background and Structure. Orphanet J Rare Dis (2020) 15(1):17. doi: 10.1186/s13023-020-1295-y 31941540 PMC6964101

[26] Shrestha S Hsu S-D Huang W-Y Huang H-Y Chen W Weng S-L. A Systematic Review of microRNA Expression Profiling Studies in Human Gastric Cancer. Cancer Med (2014) 3(4):878–88. doi: 10.1002/cam4.246 PMC4303155 24902858

[27] Blondal T Jensby Nielsen S Baker A Andreasen D Mouritzen P Wrang Teilum M. Assessing Sample and miRNA Profile Quality in Serum and Plasma or Other Biofluids. Methods (2013) 59(1):51–6. doi: 10.1016/j.ymeth.2012.09.015 23036329

[28] Scabia G Cancello R Dallanoce C Berger S Matera C Dattilo A. ICH3, a Selective Alpha7 Nicotinic Acetylcholine Receptor Agonist, Modulates Adipocyte Inflammation Associated With Obesity. J Endocrinol Invest (2020) 43(7):983–93. doi: 10.1007/s40618-020-01182-z 31965518

[29] Benjamini Y Hochberg Y. Controlling the False Discovery Rate: A Practical and Powerful Approach to Multiple Testing. J R Stat Soc Ser B (1995) 57(1):289–300. doi: 10.1111/j.2517-6161.1995.tb02031.x

[30] Eden E Navon R Steinfeld I Lipson D Yakhini Z. GOrilla: A Tool for Discovery and Visualization of Enriched GO Terms in Ranked Gene Lists. BMC Bioinformatics (2009) 10:48. doi: 10.1186/1471-2105-10-48 19192299 PMC2644678

[31] Duan Z-Y Cai G-Y Li J-J Bu R Wang N Yin P. U6 can be Used as a Housekeeping Gene for Urinary Sediment miRNA Studies of IgA Nephropathy. Available at: www.nature.com/scientificreports/. 10.1038/s41598-018-29297-7 PMC6052115 30022109

[32] Guillín-Amarelle C Sánchez-Iglesias S Castro-Pais A Rodriguez-Cañete L Ordóñez-Mayán L Pazos M. Type 1 Familial Partial Lipodystrophy: Understanding the Köbberling Syndrome. Endocrine (2016) 54(2):411–21. doi: 10.1007/s12020-016-1002-x 27473102

[33] Varlet A-A Helfer E Badens C. Molecular and Mechanobiological Pathways Related to the Physiopathology of FPLD2. Cells (2020) 9(9). doi: 10.3390/cells9091947 PMC7565540 32842478

[34] Hamam D Ali D Vishnubalaji R Hamam R Al-Nbaheen M Chen L. microRNA-320/RUNX2 Axis Regulates Adipocytic Differentiation of Human Mesenchymal (Skeletal) Stem Cells. Cell Death Dis (2014) 5(10):1947. doi: 10.1038/cddis.2014.462 PMC4237271 25356868

[35] Srinivasa S Garcia-Martin R Torriani M Fitch KV Carlson AR Kahn CR. Altered Pattern of Circulating miRNAs in HIV Lipodystrophy Perturbs Key Adipose Differentiation and Inflammation Pathways. JCI Insight (2021) 6(18). doi: 10.1172/jci.insight.150399 PMC8492307 34383714

[36] Del Vecchio G De Vito F Saunders SJ Risi A Mannironi C Bozzoni I. RNA-Binding Protein HuR and the Members of the miR-200 Family Play an Unconventional Role in the Regulation of *C-Jun* mRNA. RNA (2016) 22(10):1510–21. doi: 10.1261/rna.057588.116 PMC5029450 27473170

[37] Du H Zhao Y Yin Z Wang DW Chen C. The Role of miR-320 in Glucose and Lipid Metabolism Disorder-Associated Diseases. Int J Biol Sci (2021) 17(2):402–16. doi: 10.7150/ijbs.53419 PMC7893589 33613101

[38] McCreight JC Schneider SE Wilburn DB Swanson WJ. Evolution of microRNA in Primates. PloS One (2017) 12(6):e0176596. doi: 10.1371/journal.pone.0176596 28640911 PMC5480830

[39] Herbst KL Tannock LR Deeb SS Purnell JQ Brunzell JD Chait A. Kobberling Type of Familial Partial Lipodystrophy: An Underrecognized Syndrome. Diabetes Care (2003) 26(6):1819–24. doi: 10.2337/diacare.26.6.1819 12766116

[40] Tchkonia T Morbeck DE Von Zglinicki T Van Deursen J Lustgarten J Scrable H. Fat Tissue, Aging, and Cellular Senescence. Aging Cell (2010) 9(5):667–84. doi: 10.1111/j.1474-9726.2010.00608.x PMC2941545 20701600

[41] Donato AJ Henson GD Hart CR Layec G Trinity JD Bramwell RC. The Impact of Ageing on Adipose Structure, Function and Vasculature in the B6D2F1 Mouse: Evidence of Significant Multisystem Dysfunction. J Physiol (2014) 592(18):4083–96. doi: 10.1113/jphysiol.2014.274175 PMC4198016 25038241

[42] Li W Ding X Wang S Xu L Yin T Han S. Downregulation of Serum Exosomal miR-320d Predicts Poor Prognosis in Hepatocellular Carcinoma. J Clin Lab Anal (2020) 34(6):e23239. doi: 10.1002/jcla.23239 32125733 PMC7307335

[43] Valle-Martos R Valle M Martos R Cañete R Jiménez-Reina L Cañete MD. Liver Enzymes Correlate With Metabolic Syndrome, Inflammation, and Endothelial Dysfunction in Prepubertal Children With Obesity. Front Pediatr (2021) 16:9. doi: 10.3389/fped.2021.629346 PMC7921725 33665176

[44] Maffei M Giordano A. Leptin, the Brain and Energy Homeostasis: From an Apparently Simple to a Highly Complex Neuronal System. Rev Endocr Metab Disord (2021) 23:87–101. doi: 10.1007/s11154-021-09636-2 33822303

[45] Lim K Haider A Adams C Sleigh A Savage DB. Lipodistrophy: A Paradigm for Understanding the Consequences of “Overloading” Adipose Tissue. Physiol Rev (2021) 101(3):907–93. doi: 10.1152/physrev.00032.2020 33356916

[46] Ruiz-Ojeda FJ Wang J Bäcker T Krueger M Zamani S Rosowski S. Active Integrins Regulate White Adipose Tissue Insulin Sensitivity and Brown Fat Thermogenesis. Mol Metab (2021) 45. doi: 10.1016/j.molmet.2020.101147 PMC7808956 33359386

[47] Kechagia JZ Ivaska J Roca-Cusachs P. Integrins as Biomechanical Sensors of the Microenvironment. Nat Rev Mol Cell Biol (2019) 20(8):457–73. doi: 10.1038/s41580-019-0134-2 31182865

[48] Gao H Guo Y Yan Q Yang W Li R Lin S. Lipoatrophy and Metabolic Disturbance in Mice With Adipose-Specific Deletion of Kindlin-2. JCI Insight (2019) 4(13). doi: 10.1172/jci.insight.128405 PMC6629244 31292295

[49] Adler-Wailes DC Guiney EL Koo J Yanovski JA. Effects of Ritonavir on Adipocyte Gene Expression: Evidence for a Stress-Related Response. Obesity (2008) 16(10):2379–87. doi: 10.1038/oby.2008.350 PMC2614385 18719645

[50] Kolta S Flandre P Ngo Van P Cohen-Codar I Valantin M-A Pintado C. Fat Tissue Distribution Changes in HIV-Infected Patients Treated With Lopinavir/Ritonavir. Results of the MONARK Trial. Curr HIV Res (2011) 9(1). doi: 10.2174/157016211794582687 21198431

[51] Altay C Secil M Demir T Atik T Akinci G Ozdemir Kutbay N. Determining Residual Adipose Tissue Characteristics With MRI in Patients With Various Subtypes of Lipodystrophy. Diagn Interv Radiol (2017) 23(6):428–34. doi: 10.5152/dir.2017.17019 PMC5669542 29044029

[52] Lee PL Tang Y Li H Guertin DA. Raptor/mTORC1 Loss in Adipocytes Causes Progressive Lipodystrophy and Fatty Liver Disease. Mol Metab (2016) 5(6):422–32. doi: 10.1016/j.molmet.2016.04.001 PMC4877665 27257602

