# Supplementary material for: Circulating Levels of MiRNAs From 320 Family in Subjects With Lipodystrophy: Disclosing Novel Signatures of the Disease
Source: Front Endocrinol (Lausanne). 2022 Jun 6;13:866679. doi: 10.3389/fendo.2022.866679 (PMC9207177; doi:10.3389/fendo.2022.866679)
Supplement: Supplementary file 1 [file DataSheet_1.docx]

Supplementary Material

**Supplementary Figure 1**

**Supplementary Figure 1. Delta Ct values of cmiRs 320.** Bar graphs representing average levels of circulating miR-320a-3p, miR-320b, miR-320c, miR-320d, and miR-320e in LD (n = 32) *versus* HC (n = 23). Each asterisk denotes a pairwise comparison of 2 groups. Data, expressed as mean ± SEM. **P* < 0.05, ***P* < 0.01, *****P* < 0.0001, by Student’s t test or Mann-Whitney as appropriate.

**Supplementary Figure 2
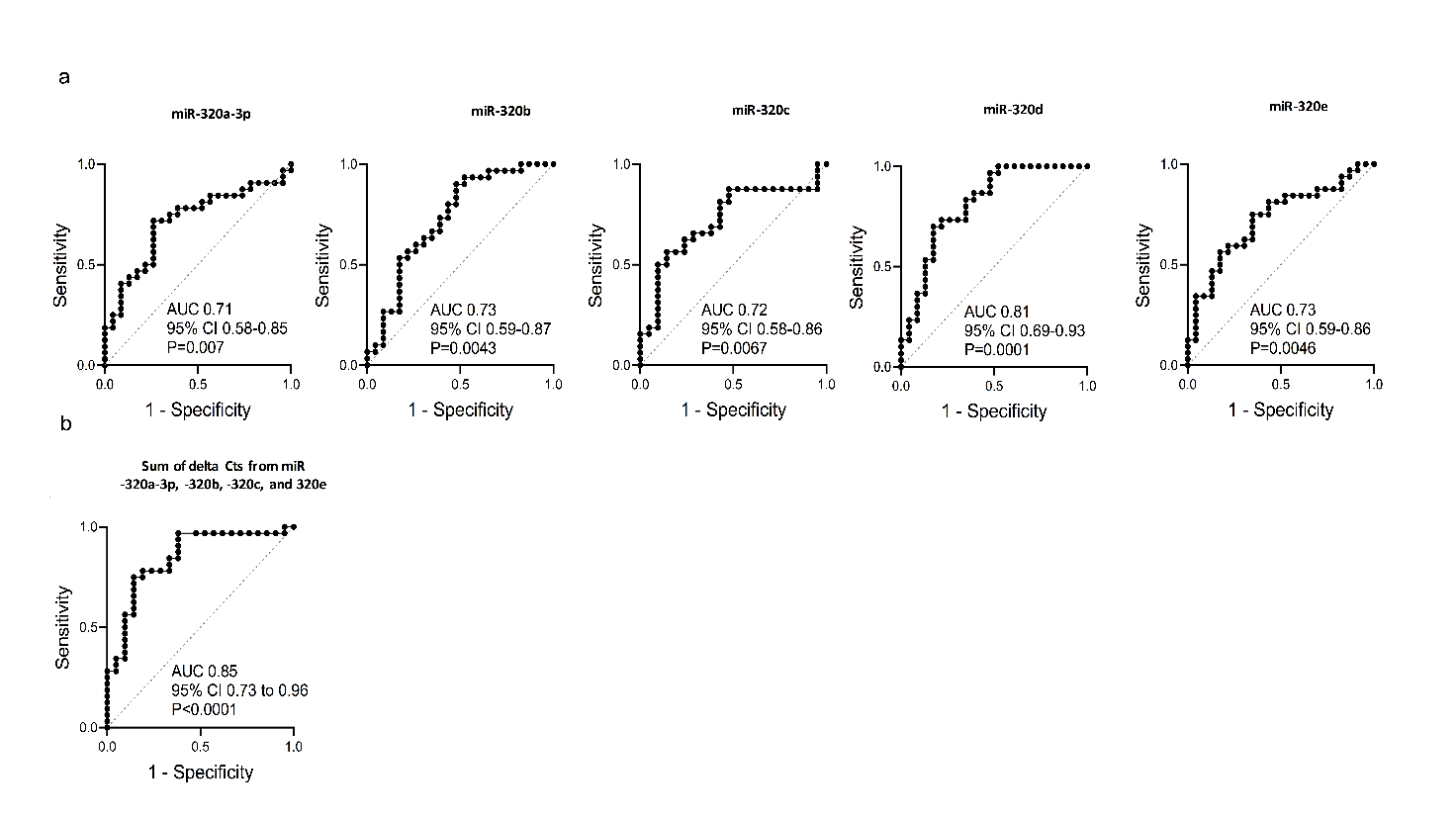
**

**Supplementary Figure 2. Receiver Operating Characteristic (ROC) curves of differentially expressed cmiRs in HC *versus* LD.** ROC curves of miR-320a-3p, miR-320b, miR-320c, miR-320d, miR-320e (a) and of miR-320a-3p+miR-320b+miR-320c+miR-320e (b). Area under the curve (AUC), confidence interval (CI) and P are shown.

**Supplementary Table 1.** Results of pairwise comparisons for cmiRs 320 in the different LD subtypes

|  | **APL vs HC** | **CGL vs HC** | **FPL vs HC** | **FPLD2 *vs* HC** | **FPLD1 *vs* Adult HC** |
| --- | --- | --- | --- | --- | --- |
| **320a-3p** | ns | P=0.0007  **↑↑↑** | P=0.055  ↑ | P=0.0042  **↑↑** | ns |
| **320b** | P=0.026  **↑** | P=0.029  **↑** | P=0.031  **↑** | P=0.0074  **↑↑** | ns |
| **320c** | ns | P=0.009  **↑↑** | P=0.029  **↑** | P=0.0003  **↑↑↑** | ns |
| **320d** | P=0.009  **↓↓** | P=0.003  **↓↓** | P=0.001  **↓↓↓** | P=0.007  **↓↓** | ns |
| **320e** | P=0.041  **↑** | P=0.009  **↑↑** | P=0.0194  **↑** | P=0.0004  **↑↑↑** | ns |

P value refers to Student’s t test or Mann-Whitney, as appropriate.

Arrows indicate an increase (upward arrows ↑) or a decrease (downward arrows ↓) of miRNAs in LD compared to HC. ↑P=0.05, **↑**or**↓** P<0.05, **↑↑** or **↓↓** P<0.01, **↑↑↑** or **↓↓↓** P<0.001. Abbreviations: APL, acquired partial lipodystrophy; CGL, congenital generalized lipodystrophy; FPL, familial partial lipodystrophy; FPLD, familial partial lipodystrophy; HC, healthy control; LD, lipodystrophy; cmiR, circulating microRNA; ns, not significant.

**Supplementary Table 2.** Physical and clinical parameters of FPLD1 subjects and age-matched healthy controls

|  | **Adult HC**  **(N=14; F=12)** | **FPLD1**  **(N=6; F=5)** |
| --- | --- | --- |
| **Age (years)** | 46.9±3.2 | 56.3±7.5 |
| **BMI (Kg/m^2^)** | 22.0±0.6 | 28.6±2.3 |
| **Glucose (mg/dL)** | 94.2±2.3 | 110.8±8.0 |
| **TG (mg/dL)** | 88.7±13.3 | 212.0±66.3 |
| **Chol (mg/dL)** | 201.0±7.0 | 197.5±12.6 |
| **HDL-C (mg/dL)** | 75.8±3.3 | 48.3±6.8 |
| **LDL-C (mg/dL)** | 125.0±6.8 | 134.2±12.7 |
| **CRP (mg/L)** | 0.02±0.01 | 1.2±0.5 |
| **AST/GOT (U/L)** | 18.2±0.9 | 28.0±6.4 |
| **ALT/GPT (U/L)** | 14.3±1.6 | 43.8±21.7 |
| **GGT (U/L)** | 14.0±2.1 | 33.0±14.6 |

Abbreviations: HC, healthy control; FPLD, familial partial lipodystrophy; BMI, body mass index; Glucose, serum fasting glucose; TG, triglycerides; Chol, total cholesterol; HDL-C, high density lipoprotein-cholesterol; LDL-C, low density lipoprotein-cholesterol; CRP, C-reactive protein; AST, aspartate aminotransferase; GOT, glutamic oxaloacetic transaminase; ALT, alanine aminotransferase; GPT, glutamic pyruvic transaminase; GGT, gamma-glutamyl transferase. Data are expressed as mean ± SEM.

**Supplementary Table 3.** Enriched Gene Ontology biological process terms for miRNAs 320 targets

| **GO term** | **Description** | **P-value** | **FDR q-value** | **Enrichment (N, B, n, b)** | **Genes** |
| --- | --- | --- | --- | --- | --- |
| GO:0007156 | Homophilic cell adhesion via plasma membrane  Adhesion molecules | 5.97E-10 | 3.93E-6 | 6.24 (936,25,96,16) | PCDHA11, PCDHA10, PCDHA8 PCDHA7, PCDHA6, PCDHA5  PCDHA4, PCDHA3, PCDHA2 PCDHA1, PCDHA12, PCDHA13  PCDH19, PCDHAC1, PCDHAC2  ITGB1 |
| GO:0098742 | Cell-cell adhesion via  plasma membrane  adhesion molecules | 6.31E-8 | 2.08E-4 | 5.03 (936,31,96,16) | PCDHA11, PCDHA10, PCDHA8 PCDHA7, PCDHA6, PCDHA5  PCDHA4, PCDHA3, PCDHA2 PCDHA1, PCDHA12, PCDHA13  PCDH19 PCDHAC1 PCDHAC2  ITGB1 |
| GO:0007399 | Nervous system  development | 8.82E-6 | 1.93E-2 | 4.39 (936,36,83,14) | PCDHA11, PCDHA10, PCDHA8 PCDHA7, PCDHA6, PCDHA5, PCDHA4, PCDHA3, PCDHA2 PCDHA1, PCDHAC1, PCDHAC2  SERF1A, SERF1B |
| GO:0098609 | Cell-cell  adhesion | 1.66E-5 | 2.74E-2 | 3.76 (936,48,83,16) | PCDHA11, PCDHA10, PCDHA8 PCDHA7, PCDHA6, PCDHA5  PCDHA4, PCDHA3, PCDHA2 PCDHA1, PCDHA12 PCDHA13  PCDHAC1, PCDHAC2  TMEM47, ITGB1 |
| GO:0022610 | Biological  adhesion | 2.89E-5 | 3.8E-2 | 2.84 (936,69,105,22) | PCDHA11, PCDHA10, PCDHA8 PCDHA7, PCDHA6, PCDHA5  PCDHA4, PCDHA3, PCDHA2 PCDHA1, PCDHA12, PCDHA13  PCDHAC1, PCDHAC2, PCDH19  PLXNC1, CD96, TMEM47 MAGI1, CNTNAP5, KITLG, ITGB1 |
| GO:0007155 | Cell-cell adhesion | 2.89E-5 | 3.17E-2 | 2.84 (936,69,105,22) | PCDHA11, PCDHA10, PCDHA8 PCDHA7, PCDHA6, PCDHA5  PCDHA4, PCDHA3, PCDHA2 PCDHA1, PCDHA12, PCDHA13  PCDHAC1, PCDHAC2, PCDH19  CD96, TMEM47, MAGI1, CNTNAP5 |
| GO:0035162 | Embryonic  hemopoiesis | 1.33E-4 | 1.25E-1 | 124.80 (936,3,5,2) | SH2B3  KITLG |

Abbreviations: GO, gene ontology; FDR, false discovery rate; PCDHAX, protocadherin alpha X; ITGB1, integrin beta 1; SERF1A, small edrk-rich factor 1a (telomeric); SERF1B, small edrk-rich factor 1b (centromeric); TMEM47, transmembrane protein 47; MAGI1, membrane associated guanylate kinase ww and pdz domain containing 1; CNTNAP5, contactin associated protein-like 5; KITLG, kit ligand.

'P-value' is the enrichment p-value computed according to the mHG or HG model.

'FDR q-value' is the correction of the above p-value for multiple testing using the Benjamini and Hochberg (1995) method. Namely, for the ith term (ranked according to p-value) the FDR q-value is (p-value * number of GO terms) / i.

Enrichment (N, B, n, b) is defined as follows:

N - is the total number of genes

B - is the total number of genes associated with a specific GO term

n - is the number of genes in the top of the user's input list or in the target set when appropriate

b - is the number of genes in the intersection

Enrichment = (b/n) / (B/N)
